# Yin Yang-1 suppresses invasion and metastasis of pancreatic ductal adenocarcinoma by downregulating MMP10 in a MUC4/ErbB2/p38/MEF2C-dependent mechanism

**DOI:** 10.1186/1476-4598-13-130

**Published:** 2014-05-29

**Authors:** Jing-Jing Zhang, Yi Zhu, Kun-Ling Xie, Yun-Peng Peng, Jin-Qiu Tao, Jie Tang, Zheng Li, Ze-Kuan Xu, Cun-Cai Dai, Zhu-Yin Qian, Kui-Rong Jiang, Jun-Li Wu, Wen-Tao Gao, Qing Du, Yi Miao

**Affiliations:** 1 Department of General Surgery, The first Affiliated Hospital of Nanjing Medical University, Jiangsu Province Academy of Clinical Medicine, Institute of Tumor Biology, 300 Guangzhou Road, Nanjing 210029, People’s Republic of China; 2 Jiangsu Province Academy of Clinical Medicine, Institute of Tumor Biology, 300 Guangzhou Road, Nanjing 210029, People’s Republic of China; 3 The First School of Clinical Medicine, Nanjing Medical University, 140 Hanzhong Road, Nanjing 210029, People’s Republic of China

**Keywords:** Yin Yang-1, Pancreatic ductal adenocarcinoma, Metastasis, MMP10, MUC4, MEF2C

## Abstract

**Background:**

Increasing evidence indicates an important role of transcription factor Yin Yang-1 (YY1) in human tumorigenesis. However, its function in cancer remains controversial and the relevance of YY1 to pancreatic ductal adenocarcinoma (PDAC) remains to be clarified.

**Methods:**

In this study, we detected YY1 expression in clinical PDAC tissue samples and cell lines using quantitative RT-PCR, immunohistochemistry and western blotting. We also detected MUC4 and MMP10 mRNA levels in 108 PDAC samples using qRT-PCR and analyzed the correlations between YY1 and MUC4 or MMP10 expression. The role of YY1 in the proliferation, invasion and metastatic abilities of PDAC cells in vitro was studied by CCK-8 assay, cell migration and invasion assays. In vivo pancreatic tumor growth and metastasis was studied by a xenogenous subcutaneously implant model and a tail vein metastasis model. The potential mechanisms underlying YY1 mediated tumor progression in PDAC were explored by digital gene expression (DGE) sequencing, signal transduction pathways blockage experiments and luciferase assays. Statistical analysis was performed using the SPSS 15.0 software.

**Results:**

We found that the expression of YY1 in PDACs was higher compared with their adjacent non-tumorous tissues and normal pancreas tissues. However, PDAC patients with high level overexpression of YY1 had better outcome than those with low level overexpression. YY1 expression levels were statistically negatively correlated with MMP10 expression levels, but not correlated with MUC4 expression levels. YY1 overexpression suppressed, whereas YY1 knockdown enhanced, the proliferation, invasion and metastatic properties of BXPC-3 cells, both in vitro and in vivo. YY1 suppresses invasion and metastasis of pancreatic cancer cells by downregulating MMP10 in a MUC4/ErbB2/p38/MEF2C-dependent mechanism.

**Conclusions:**

The present study suggested that YY1 plays a negative role, i.e. is a tumor suppressor, in PDAC, and may become a valuable diagnostic and prognostic marker of PDAC.

## Background

Pancreatic ductal adenocarcinoma (PDAC) is one of the most deadly cancers in the world. The survival time is extremely short (6 months) and the survival rate at 5 years is very low (3%) [1]. This poor outcome is related to a lack of efficient therapeutic tools and early diagnostic markers. At the time of diagnosis, more than 80% of PDAC patients are already metastatic or have locally advanced cancer, and only about 10% to 15% of patients are considered eligible for surgical resection [2]. Chemotherapy is often either ineffective or effective only for a short duration. Further therapeutic approaches are needed in addition to conventional chemotherapy for this deadly disease. Molecular-targeted therapy, including activation of tumor suppressor genes, and/or repression of oncogenes, is an attractive therapeutic addition to regular chemotherapies. Thus, it is very important to find new therapeutic molecular targets of PDAC.

Yin Yang-1 (YY1) is a ubiquitously expressed, multifunctional zinc finger transcription factor that belongs to the GLI-Kruppel family of nuclear proteins [3]. YY1 has a fundamental role in biological and physiological processes, such as embryogenesis, cellular proliferation, DNA replication and differentiation [3-5]. YY1 can act as a transcriptional activator, repressor or initiator protein depending upon DNA binding site context or cell type [6-9]. Increasing evidence indicates an important role of YY1 in human tumorigenesis. However, the data are somewhat diverse, with some cancers showing increased YY1 expression and some showing decreased expression. For example, increased YY1 expression was observed in cancers of the prostate, ovary, colon, breast, bone, liver, lung, bladder, cervix and blood [10-19]. Reduced YY1 expression was found in some melanomas, pediatric osteosarcomas and urothelial carcinomas [15]. In addition, conflicting data exist on the survival outcome in relation to YY1 expression. In some cases, high expression of YY1 correlated with poor prognosis (such as in prostate, breast and bone cancers) [10,13,20], whereas in other situations, YY1 expression correlated with favorable outcomes (such as in ovarian cancer, colon cancer and follicular lymphoma) [11,12,21]. These studies indicated strongly the important role of YY1 in cancer development. However, the relevance of YY1 to PDAC remains to be clarified. We thus proposed to study YY1 expression in clinical PDAC samples and the potential mechanisms underlying YY1 mediated tumor progression in PDAC. In this study, we found that the expression of YY1 in PDACs was higher compared with their adjacent non-tumorous tissues and normal pancreas tissues. However, PDAC patients with high level overexpression of YY1 had better outcome than those with low level overexpression. YY1 overexpression suppressed, whereas YY1 knockdown enhanced, the proliferation, invasion and metastatic properties of BXPC-3 cells. YY1 suppresses invasion and metastasis of pancreatic cancer cells by downregulating MMP10 in a MUC4/ErbB2/p38/MEF2C-dependent mechanism. These results indicated that YY1 might become a valuable diagnostic and prognostic marker for PDAC.

## Results

### YY1 expression in PDAC tissues and cell lines

**Figure 1:**
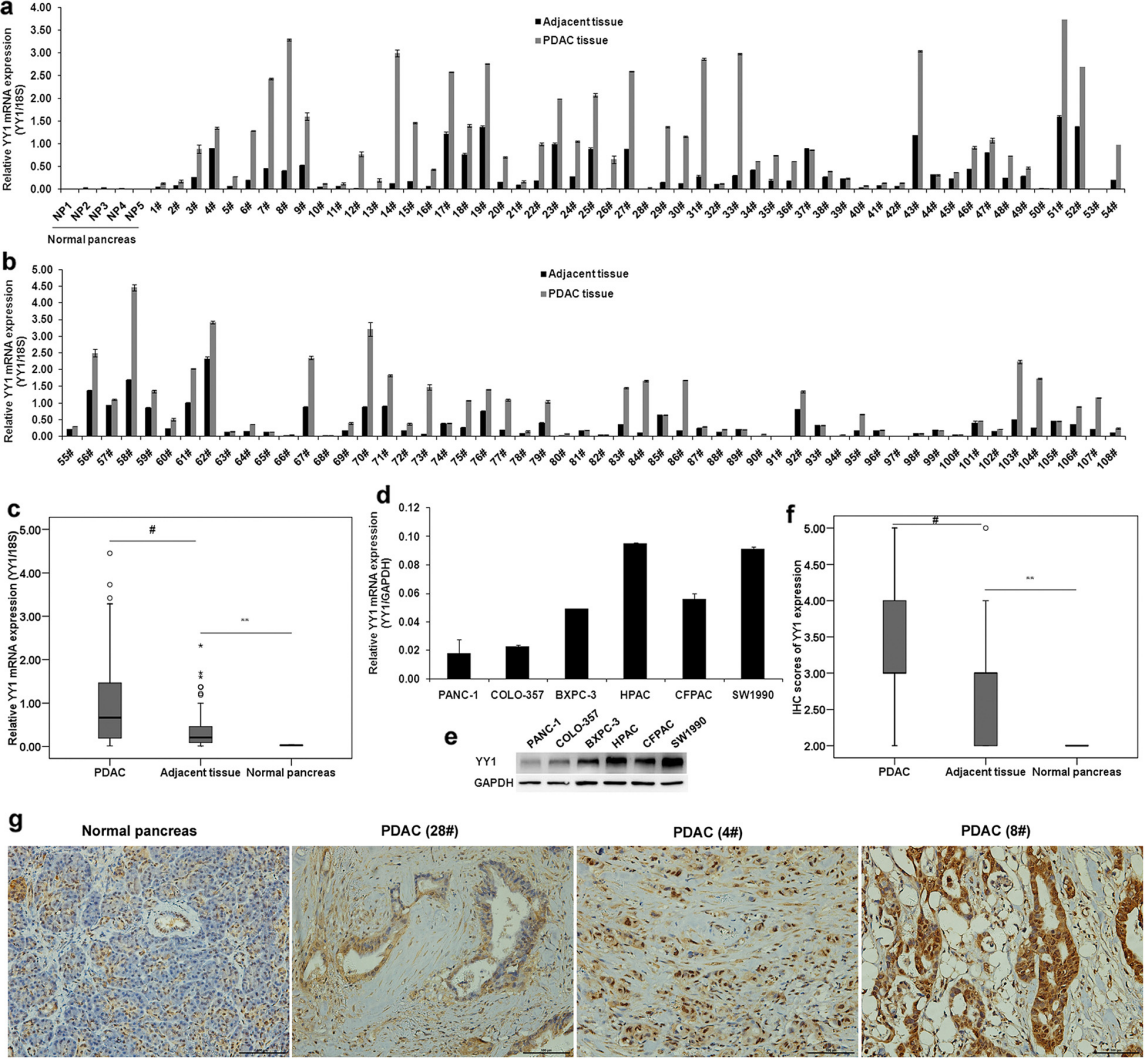
**YY1 expression in PDAC tissues and cell lines. (a, b)** qRT-PCR analysis of YY1 expression in five normal pancreas tissues and 108 pairs of PDAC tissue and adjacent tissue. YY1 expression in the normal pancreas tissues was low, but was slightly higher in the tissue adjacent to PDAC tumors and significantly higher in the PDAC tumors themselves. Of the 108 paired tissues, YY1 expression was higher in the cancer tissue than in the adjacent tissues in 86 cases (*p* < 0.05). **(c)** A boxplot showing the qRT-PCR results of YY1 mRNA expression in the 108 cancer tissue, 108 adjacent tissue and five normal pancreas tissues (mean ± SD: 1.01 ± 1.01, 0.38 ± 0.43 and 0.03 ± 0.01, respectively). The bottom and the top edges of the box mark the lower boundary and upper boundary of the 95% confidence interval (CI) for the mean. The center horizontal line is drawn at the sample mean. The center vertical lines drawn from the boxes extend to the minimum and the maximum. **(d)** qRT-PCR analysis of YY1 expression in six PDAC cell lines. **(e)** Western blotting of YY1 expression in six PDAC cell lines. **(f)** A boxplot showing the immunohistochemistry (IHC) results of YY1 protein expression in the 108 cancer tissue, 108 adjacent tissue and five normal pancreas tissues. **(g)** IHC showing the location of YY1 in PDAC samples. Scale bar, 100 μm. (** represents *p* < 0.01, # represents *p* < 0.001, compared with the control group).

We measured YY1 mRNA expression in five normal pancreas tissue samples and in 108 PDAC tissue samples with paired adjacent non-tumorous tissue using qRT-PCR. As observed in Figure 1a and b, YY1 expression in the normal pancreas tissue samples was low, but was slightly higher in the tissue adjacent to PDAC tumors and significantly higher in the PDAC tumors themselves. Of the 108 paired samples, 87 (80.6%) showed significantly higher YY1 expression in the tumor tissues compared with the adjacent tissues (*p* < 0.05). The same trend could be observed using a boxplot (Figure 1c), which showed that the mean expression level of YY1 in tumors, tumor adjacent normal tissues and normal pancreas was 1.01 ± 1.01, 0.38 ± 0.43 and 0.03 ± 0.01, respectively. We then detected YY1 mRNA and protein expression in PDAC cell lines. As observed in Figure 1d, of the six cell lines tested, HPAC and SW1990 expressed YY1 at high levels, BXPC-3 and CFPAC-1 expressed YY1 at moderate levels, and PANC-1 and COLO-357 cells showed low levels of YY1 expression. In addition, the western blotting results agreed with the results of qRT-PCR (Figure 1e). We then detected YY1 protein expression in clinical samples using immunohistochemistry. The results (Figure 1f) showed that YY1 protein expression in the normal pancreas tissue samples was low, but was slightly higher in the tissue adjacent to PDAC tumors and significantly higher in the PDAC tumors themselves, which agreed with the results of qRT-PCR. From Figure 1g, we observed that YY1 was located in the nucleus and cytoplasm of tumor cells. Moreover the YY1 RNA levels (qRT-PCR results) were statistically correlated with protein levels (IHC results) ((r = 0.854, *p* < 0.001). Taken together, our results show that YY1 is highly expressed in PDAC tissues, indicating that YY1 might be activated during pancreatic tumorigenesis and may have an important role in PDAC.

### Correlations between YY1 mRNA expression and clinicopathological or serological variables of PDAC patients

Correlations between YY1 mRNA expression and clinicopathological or serological characteristics in PDACs from all 108 patients are shown in Table S1 (Additional file 1). YY1 expression was statistically correlated with age, differentiation and TNM staging (*p* = 0.022, *p* < 0.001 and *p* = 0.008, respectively). Elevated YY1 expression is enriched in well-differentiated tumors, in lower TNM grades and in tumors in young patients. No significant correlations were identified between YY1 expression and other variables.

### YY1 is an independent predictor of favorable outcome of PDAC patients

**Figure 2:**
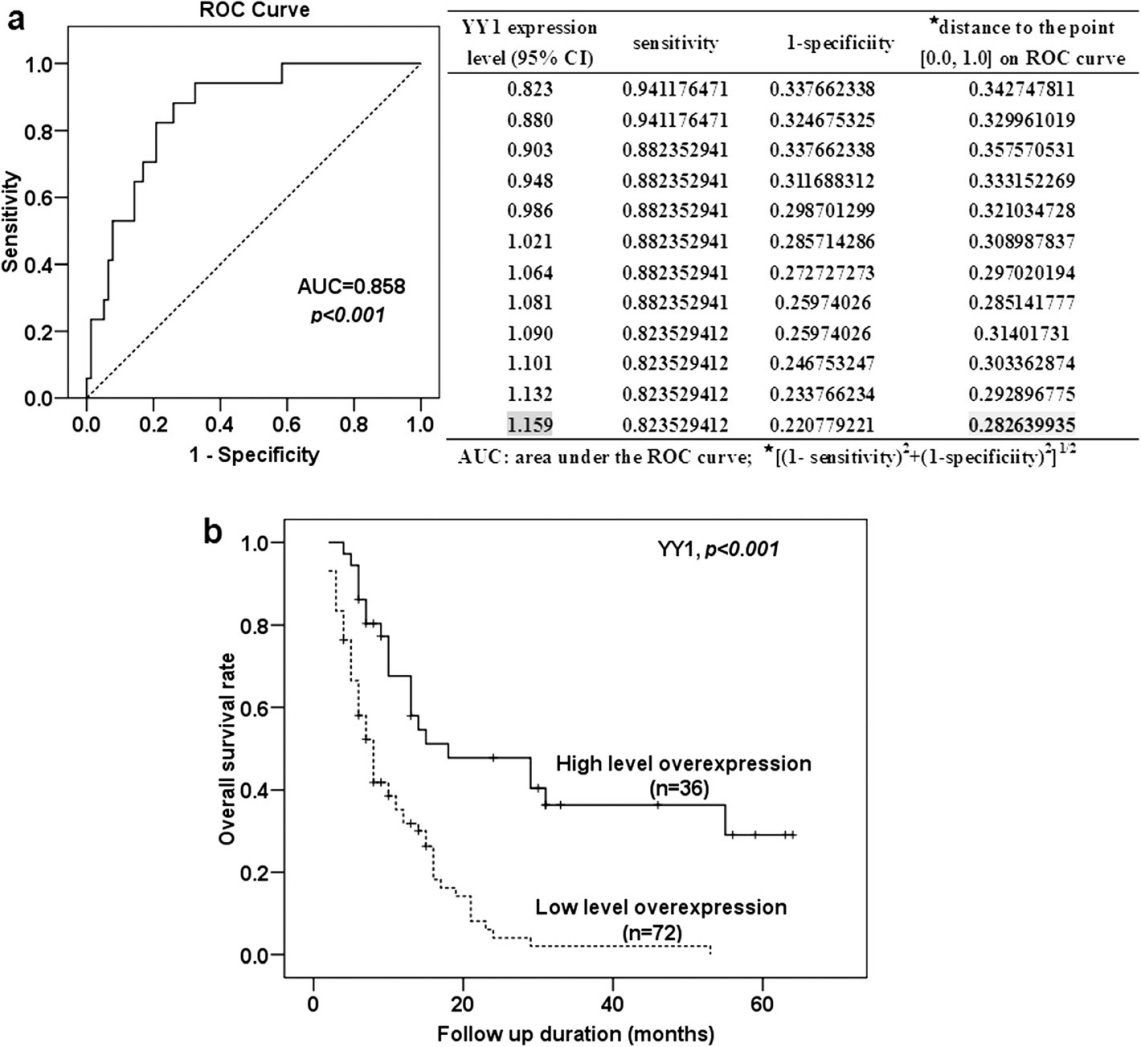
**Correlation between YY1 expression and patient survival. (a)** ROC curve for YY1 expression and cut-off value selection for high and low level YY1 expression. **(b)** Kaplan-Meier survival curves in 108 patients with PDAC according to their YY1 mRNA expression status. The *p* value was calculated by the Log-rank test.

One hundred and eight PDAC patients were enrolled in the survival analysis. Eighty-four patients died, while the remaining 24 patients were alive at the last follow up (31 March 2013). To determine the YY1 expression level cut-off value for survival analysis, the patients were divided into two groups based on the length of overall survival: short-term survivors (survival period < 24 months) and long-term survivors (≥24 months). The threshold value of 1.159 was chosen as the cut-off value for high and low level overexpression of YY1, because 1.159 (within 95% confidence interval (CI), 0.821-1.208, of YY1 mRNA expression) was on the ROC curve closest to (0.0, 1.0). This maximized both sensitivity and specificity for the survival outcome (Figure 2a). The area under the ROC curve (AUC) was 0.858 (95% CI, 0.773-0.943, *p* < 0.001).

The Kaplan-Meier survival curves showed that patients with high level (≥1.159) overexpression of YY1 had longer post-operative survival than those with low level (<1.159) overexpression (Figure 2b, *p* < 0.001, log rank test). The two-year survival of patients with high-level overexpression of YY1 was 37.9%. This was significantly higher than that of patients with low-level overexpression (3.1%).

Meanwhile, based on 0.10 as the cut off *p*-value, seven factors (location of tumor, tumor differentiation, TNM staging, serum CA19-9, serum CA50, serum CEA and YY1 expression) were selected from the univariate analysis data (Additional file 2: Table S2) for forward and backward stepwise multivariate Cox’s proportional hazard analysis. Table 1 shows that tumor differentiation (*p* = 0.022, *p* = 0.007, respectively), serum CA19-9 (*p* = 0.036, *p* = 0.028, respectively) and YY1 expression (*p* = 0.004, *p* = 0.032, respectively) were significant independent risk factors for predicting the prognosis of PDAC patients. These results indicated that higher overexpression of YY1 predicted better outcome in PDAC patients, although YY1 expression increased in PDAC tissues.

**Table 1:** Multivariate analysis of prognostic factors in PDAC patients (n = 108)

**Variable**	**Forward stepwise**^**a**^		**Backward stepwise**^**b**^	
	**HR**^**c **^**(95% CI**^**d**^**)**	***p***	**HR (95% CI)**	***p***
Location of tumor		0.074		0.004^*^
Head	-		1	
Body and tail	-		2.178 (1.274-3.725)	
Differentiation		0.022^*^		0.007^*^
Well	1		1	
Moderate	1.560 (0.681-3.572)	0.293	1.726 (0.752-3.961)	0.198
Poor	3.775 (1.302-10.943)	0.014^*^	4.807 (1.661-13.913)	0.004^*^
TNM staging		0.089		0.015^*^
IA + IB	-		1	
IIA	-	0.804	2.694 (1.163-6.240)	0.021^*^
IIB	-	0.666	2.892 (1.306-6.404)	0.009^*^
III + IV	-	0.039	4.277 (1.746-10.476)	0.001^*^
Serum CA19-9 (kU/L)		0.036^*^		0.028^*^
≤ 39	1		1	
> 39	1.803 (1.038-3.130)		1.881 (1.071-3.304)	
YY1		0.004^*^		0.032^*^
Low level overexpression	1		1	
High level overexpression	0.416 (0.229-0.756)		0.511 (0.277-0.944)	

^a^Forward stepwise, the parameters entered one by one, each time the parameter with the smallest *p*-value was entered, until the parameters not entered have no significance; ^b^Backward stepwise, all parameters entered the first-time, and then removed one by one, each time removing the parameter with the largest *p*-value, until all of the parameters retained have significance; ^c^HR, hazard ratio; ^d^95% CI, 95% confidence interval; ^*^p < 0.05.

### Generation of stable transfected cells

**Figure 3:**
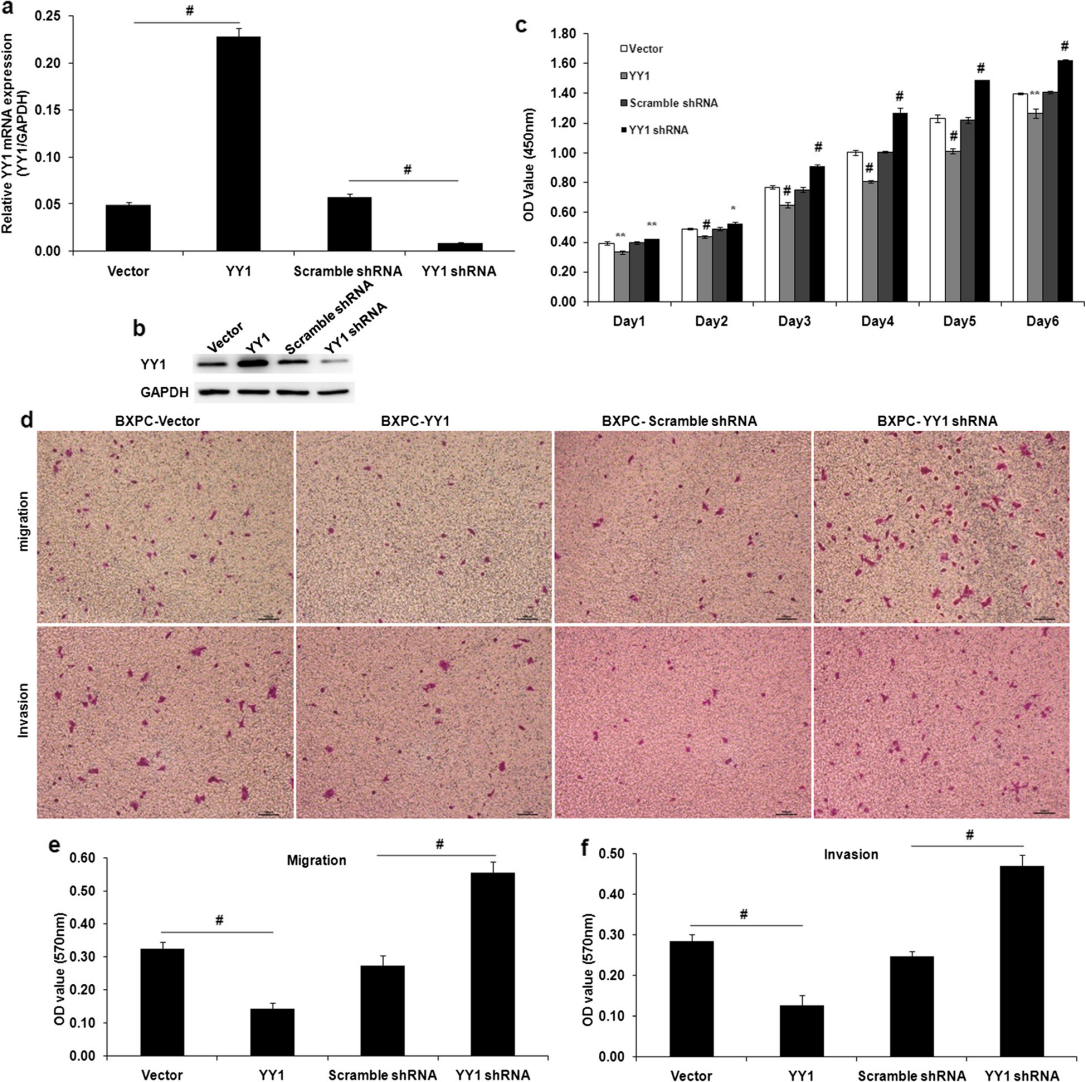
**Effects of YY1 on the proliferation, migration and invasion of BXPC-3 cells in vitro. (a)** Measurement of YY1 expression in stably transfected BXPC-3 cells using qRT-PCR. BXPC-YY1 indicates YY1overexpressing BXPC-3 cells; BXPC-Vector indicates BXPC-3 cells transfected with a control vector; BXPC-YY1 shRNA indicates YY1 knockdown BXPC-3 cells; BXPC-Scramble shRNA indicates BXPC-3 cells transfected with a vector expressing Scramble shRNA. **(b)** Measurement of YY1 expression in stably transfected BXPC-3 cells using western blotting. The results are similar to those seen in the qRT-PCR analysis. **(c)** CCK-8 assays were performed. OD values from four independent experiments were assessed. **(d)** Cell migration and invasion assays were performed. The upper chambers were seeded with various cell lines. The membranes of the chambers were stained with 0.1% crystal violet. Scale bar, 100 μm. **(e, f)** Quantification of the data from Figure 3d. OD values from three independent experiments were assessed. (*represents *p* < 0.05, **represents *p* < 0.01, # represents *p* < 0.001, compared with the control group).

To further investigate the roles of YY1 in PDAC, we overexpressed and silenced YY1 expression. BXPC-3 cells with a moderate level of YY1 expression were chosen for these experiments. We constructed YY1 overexpression and YY1 knockdown lentiviruses to infect BXPC-3 cells and selected stably infected cells. We confirmed the YY1 expression levels using qRT-PCR and western blotting (Figure 3a and 3b).

### YY1 suppresses proliferation of BXPC-3 cells in vitro

To investigate the effect of YY1 overexpression or knockdown on BXPC-3 cell proliferation in vitro, we employed the Cell Count Kit-8 (CCK-8) assay to evaluate the effects of YY1 on cell growth. As shown in Figure 3c, the curves of growth according to absorbance indicated that YY1 overexpression significantly decreased the OD value compared with the control group, while YY1 knockdown significantly increased the OD value compared with the control group. These results suggest a role for YY1 in the negative regulation of BXPC-3 cell proliferation.

### YY1 suppresses migration and invasion of BXPC-3 cells in vitro

YY1 overexpression significantly suppressed the migration of BXPC-3 cells (BXPC-YY1 *vs*. BXPC-Vector, 0.142 ± 0.018 vs. 0.324 ± 0.022, *p* < 0.001) (Figure 3d and 3e). By contrast, YY1 knockdown promoted the migration of BXPC-3 cells (BXPC-YY1 shRNA *vs*. BXPC-Scramble shRNA, 0.555 ± 0.033 *vs*. 0.271 ± 0.032, *p* < 0.001) (Figure 3d and 3e). Cell invasion assays showed that YY1 overexpression significantly decreased the invasion of BXPC-3 cells (BXPC-YY1 *vs*. BXPC-Vector, 0.1272 ± 0.024 *vs*. 0.285 ± 0.016, *p* < 0.001), while YY1 knockdown enhanced the invasion of BxPC-3 cells (BXPC-YY1 shRNA *vs*. BXPC-Scramble shRNA, 0.470 ± 0.028 *vs*. 0.247 ± 0.013, *p* < 0.001) (Figure 3d and 3f). These results suggest a role for YY1 in the negative regulation of BXPC-3 cell migration and invasion.

### YY1 suppresses tumor growth and metastasis of BXPC-3 cells in vivo

**Figure 4:**
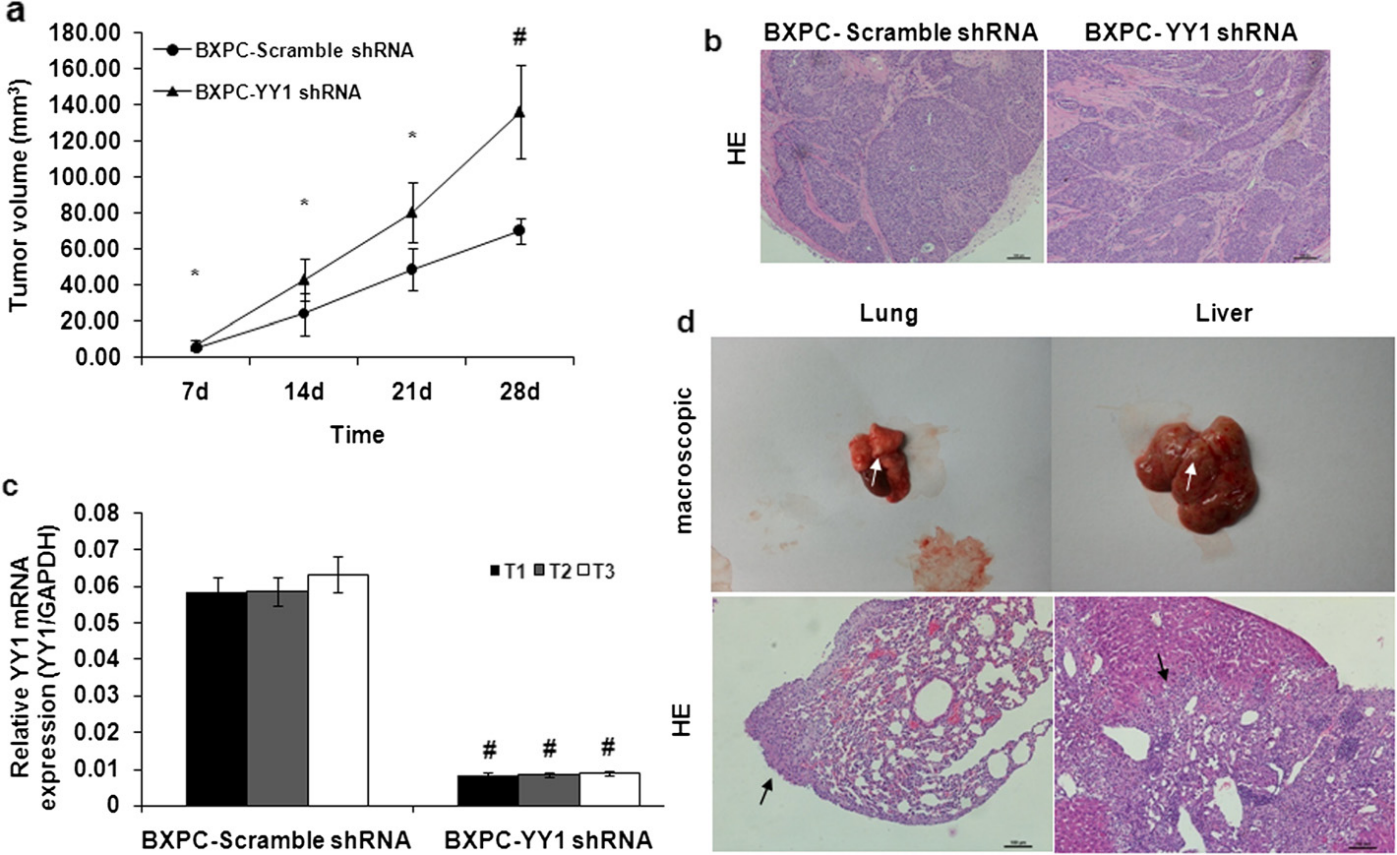
**Effects of YY1 on the tumor growth and metastasis of BXPC-3 cells in vivo. (a)** BXPC-Scramble shRNA and BXPC-YY1 shRNA cells were bilaterally injected into the flank region of the mice (1.5 × 10^6^ cells/100 μl per flank). After injection, the length and width of the tumors were measured every week. The volume of the tumors was calculated using the formula (width^2^ × length)/2. The data are presented as means ± SD of 16 tumors for each group. **(b)** HE staining of the subcutaneous tumors excised four weeks later. Scale bar, 100 μm. **(c)** Three tumors from each group were resected, RNA was extracted and YY1 expression was measured using qRT-PCR. **(d)** BXPC-Vector and BXPC-YY1 cells (1.5 × 10^6^ cells/100 μl) were separately injected into the tail vein of each mouse. Four weeks later, lung and liver metastases were evaluated by macroscopic observation and by histomorphology under microscopy. Scale bar, 100 μm. The arrows indicate the metastases. (* represents *p* < 0.05, # represents *p* < 0.001, compared with the control group).

To further study the effects of YY1 on tumorigenicity and metastasis of PDAC, in vivo experiments were performed via the subcutaneous or tail vein injection of transduced cells into BALB/cA-nu nude mice. After injection, in the xenograft tumor model, we measured the size of the growing tumors weekly for four weeks, after which the mice were euthanized. The tumor sizes of the BXPC-YY1 shRNA group were significantly larger than the BXPC-Scramble shRNA group (Figure 4a). The subcutaneous tumors were excised, stained using HE stain and RNA extracted from them to confirm that the stable knockdown of YY1 was maintained (Figure 4b and 4c). In the tail vein metastasis model, lung or liver metastases were observed in only two of the eight mice injected with BXPC-YY1 cells. By contrast, metastases were observed in seven of the eight mice injected with BXPC-Vector cells (Table 2, Figure 4d). The statistical analysis of the data showed a significant difference between the BXPC-YY1 cells and BXPC-Vector cells (*p* = 0.0406). In addition, in the xenograft models, no metastasis was found in the BXPC-Scramble shRNA control group, whereas there were two lung metastasis in the BXPC-YY1 shRNA group. However, there was no significant difference between the BXPC-YY1 shRNA group and BXPC-Scramble shRNA group (*p* = 0.4667). The small number of samples in the experiment means that the result needs further confirmation. These data indicate that knockdown of YY1 significantly promotes tumor growth, and overexpression of YY1 significantly suppresses metastasis of BXPC-3 cells in vivo, indicating that YY1 has a negative role in PDAC tumorigenicity and metastasis.

**Table 2:** Metastasis in the xenogenous subcutaneously implant model and tail vein metastasis model

**Number of animals**	**Xenogenous subcutaneously implant model**		**Tail vein metastasis model**	
	**BXPC-scramble shRNA**	**BXPC-YY1 shRNA**	**BXPC-Vector**	**BXPC-YY1**
Metastasis	0	2	7	2
No metastasis	8	6	1	6
Lung metastasis	0	2	7	2
Liver metastasis	0	0	3	1

### Differentially expressed genes (DEGs) between YY1 knockdown BXPC-3 cells and control cells

**Figure 5:**
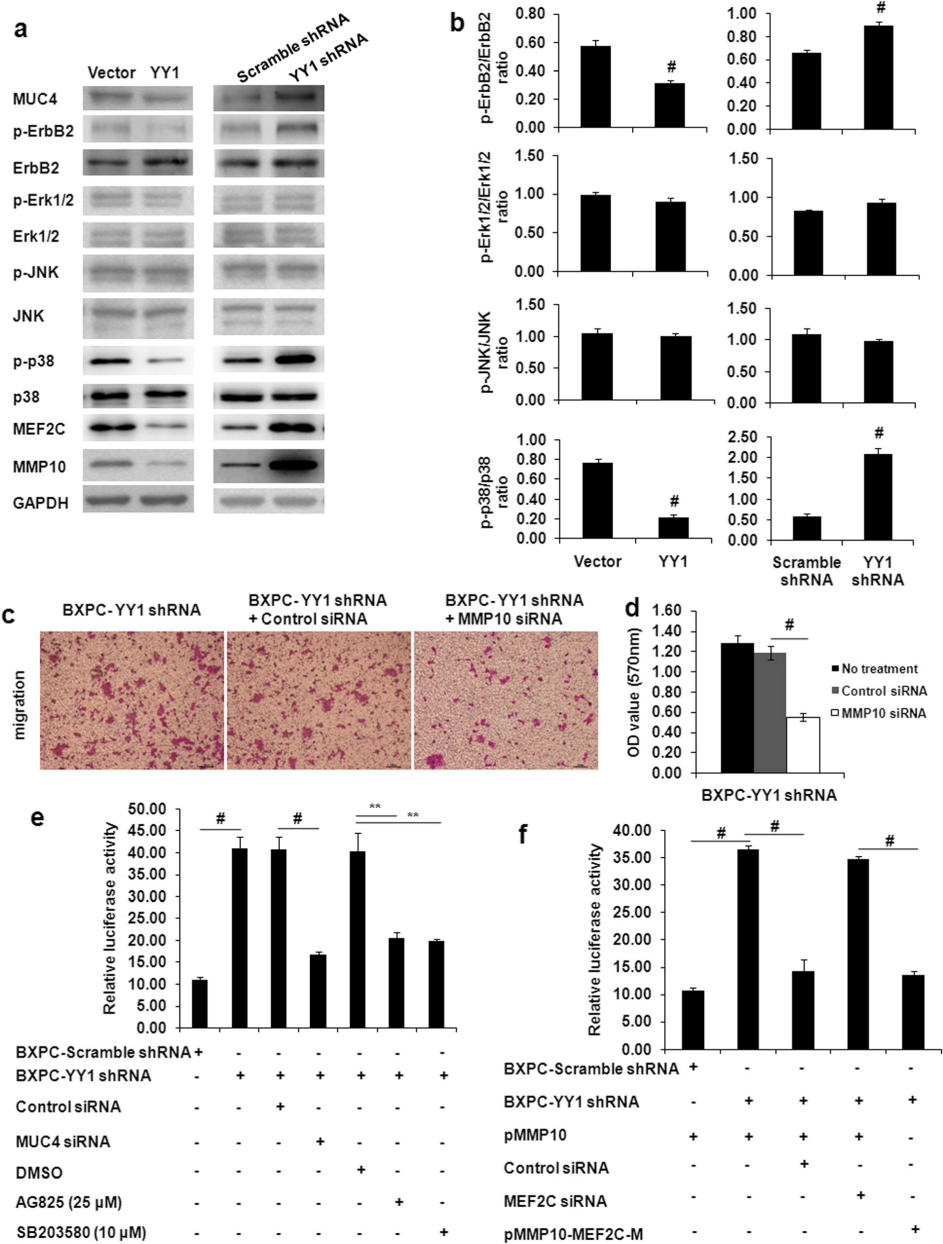
**Mechanisms involved in the tumor suppression role of YY1. (a)** Effects of YY1 on MUC4, ErbB2, MEF2C, MMP10 and MAPK (Erk1/2, JNK and p38) signaling pathways were assayed by western blotting. BXPC-YY1 indicates YY1overexpressing BXPC-3 cells; BXPC-Vector indicates BXPC-3 cells transfected with a control vector; BXPC-YY1 shRNA indicates YY1 knockdown BXPC-3 cells; BXPC-Scramble shRNA indicates BXPC-3 cells transfected with a vector expressing Scramble shRNA. GAPDH was used as the internal control. **(b)** Bands were quantified by densitometry. Histograms of the ratio (phosphorylated/constitutive form) for Erk1/2, JNK and p38 MAPK kinases are shown. **(c)** BXPC-YY1 shRNA cells were transfected with MMP10 siRNA or negative control siRNA. 12 h after transfection, cell migration assays were performed. The upper chambers were seeded with various cell lines. The membranes of the chambers were stained with 0.1% crystal violet. Scale bar, 100 μm. **(d)** Quantification of the data from Figure 5c. OD values from three independent experiments were assessed. **(e)** Luciferase activities of MMP10 promoter-transfected BXPC-Scramble shRNA or BXPC-YY1 shRNA cells treated with MUC4 siRNA or various inhibitors of signal transduction molecules. MUC4 blockage, the ErbB2 inhibitor (AG825, 25 μM) and the p38 inhibitor (SB203580, 10 μM) significantly inhibited YY1 knockdown-stimulated luciferase activity in BXPC-YY1 shRNA cells transfected with the MMP10 promoter. **(f)** The YY1 knockdown-stimulated luciferase activity could be significantly inhibited by MEF2C blockage. Luciferase activity of MMP10 promoter in BXPC-YY1 shRNA cells was significantly decreased when the presumed MEF2C binding site (nucleotides −881 to −890) was mutated. (*represents *p* < 0.05, **represents *p* < 0.01, # represents *p* < 0.001, when compared to the control group).

To identify potential YY1 target genes in BXPC-3 cells that might reveal the mechanism by which YY1 controls invasion and metastasis of PDAC, we used DGE to compare the gene expression differences between YY1 knockdown BXPC-3 cells and control cells. The expression abundance of tag-mapped genes in the data sets was analyzed by counting the number of TPM clean tags. We performed two biological replicates of the DGE sequencing. Analysis of the first biological replicate revealed 13,603 and 13,793 genes for YY1 knockdown BXPC-3 cells and control cells, respectively. Analysis of the second biological replicate revealed 13,924 and 14,044 genes for YY1 knockdown BXPC-3 cells and control cells, respectively. After taking the average of two biological replicates, we performed differential gene expression analysis. The results showed that there were 561 genes (about 4%) that were differentially expressed between the two groups (Additional file 3: Table S3). The threshold for judging the statistical significance of gene expression was FDR ≤ 0.001 and the absolute value of log2 ratio ≥ 1. Of the 561 genes, 136 were upregulated and 425 were downregulated in YY1 knockdown BXPC-3 cells. Of particular interest were the genes encoding matrix metalloproteinase 10 (MMP10), also referred to as stromelysin 2, which was upregulated by 3.3-fold, and its inhibitor tissue inhibitor of metalloproteinase 2 (TIMP2), which was downregulated by 3.2-fold. MMP10 is a secreted proteinase that degrades the extracellular matrix, and TIMP2 inhibits MMP10 activity [22]. Thus, the upregulation of MMP10 and concomitant downregulation of TIMP2 would be predicted to disrupt cell-cell adhesion and promote invasion and metastasis of YY1 knockdown BXPC-3 cells. In addition, among the 205 upregulated genes, the pancreatic cancer-related gene MUC4 was upregulated by 4.0-fold, indicating that YY1 may negatively regulate MUC4 gene expression. This result is consistent with our previous study [23], which demonstrated that YY1 inhibits MUC4 gene transcription and expression by binding to the inhibitory element in the MUC4 promoter. Previous studies showed that MUC4 and ErbB2 might interact physically and transduce signals intracellularly, thus promoting the migratory and metastatic potential of pancreatic cancer cells [24-26]. In addition, the myocyte-specific enhancer factor 2C (MEF2C), a direct activator of MMP10 transcription [27], was upregulated by 3.5-fold. The changes in expression of these four genes in YY1 knockdown BXPC-3 cells were validated by qRT-PCR. The qRT-PCR data for these genes were consistent with those obtained by DGE expression profiling (Additional file 4: Figure S1). Western blotting showed that the protein levels of MUC4, p-ErbB2, MEF2C and MMP10 were increased in YY1 knockdown BXPC-3 cells compared with control cells (Figure 5a). Therefore, we presume that YY1 suppresses invasion and metastasis of pancreatic ductal adenocarcinoma by downregulating MMP10 in a MUC4/ErbB2/MEF2C-dependent mechanism.

### GO functional enrichment analysis for differentially expressed genes (DEGs)

To gain an insight into the possible mechanisms of the differences in gene expression between YY1 knockdown BXPC-3 cells and control cells, GO enrichment analysis of the DEGs was performed. All DEGs were assigned functionally into three groups: (1) biological process; (2) cellular component; (3) molecular function. Within biological process, cellular process and metabolic process represented the most abundant GO terms. Most DEGs that corresponded to cellular component were involved in cell and cell part. Binding and catalytic activity were the most prevalent in molecular function (Additional file 5: Figure S2).

### Correlations between YY1 and MUC4 or MMP10 mRNA expression in PDAC tissues

We also detected the MUC4 and MMP10 mRNA levels in 108 PDAC samples using quantitative RT-PCR and then analyzed the correlations between the expressions of YY1 and MUC4 or MMP10. The results showed that YY1 expression levels were statistically negatively correlated with MMP10 expression levels (r = −0.472, *p* < 0.001), but not statistically correlated with MUC4 expression levels (r = −0.158, *p* = 0.102). These results, combined with those of the in vitro experiments, suggested that YY1 may negatively regulate the expression of MMP10, thereby inhibiting metastasis of PDAC.

### YY1 regulates MMP10 to affect the migrating and invasive abilities of BXPC-3 cells

To address whether YY1 knockdown-mediated tumor invasion is dependent on MMP10 activity, we used siRNA experiments to knockdown MMP10 expression in vitro. As shown in Figure 5c and 5d, cell migration was significantly reduced when the MMP10 activity was blocked by siRNA in YY1 knockdown BXPC-3 cells (BXPC-YY1 shRNA + MMP10 siRNA *vs*. BXPC-YY1 shRNA + Control siRNA, 0.552 ± 0.040 *vs*. 1.188 ± 0.065, *p* < 0.001). Invasion experiments also showed similar results (data not shown). These results suggested that MMP10 plays an important role in YY1 knockdown-mediated cell invasive and migratory behaviors.

### YY1 knockdown upregulates MMP10 by activating the MUC4/ErbB2/p38 MAPK signal transduction pathway

The impact of YY1 on the major pathways of intracellular signaling was studied by western blot for: ErbB2, MAPKs (Erk1/2, JNK and p38), Akt, and Focal Adhesion Kinase (FAK) (Figure 5a, 5b and Additional file 6: Figure S3). Phosphorylation of ErbB2 and phosphorylation of p38 MAPK decreased in YY1 overexpression BXPC-3 cells, whereas they increased in YY1 knockdown BXPC-3 cells, compared with control cells, indicating activation of ErbB2 and p38 MAPK pathways subsequent to YY1 silencing. Erk1/2 and JNK MAPK pathways did not appear to be significantly affected by either YY1 overexpression or YY1 knockdown (Figure 5a and 5b). Similarly, Akt and FAK pathways were not significantly modified (Additional file 6: Figure S3).

We next evaluated whether the YY1 knockdown-induced transduction molecules enhanced MMP10 expression at the level of transcriptional using luciferase reporter activity assays. YY1 knockdown significantly increased the luciferase activities of MMP10 and this effect could be suppressed by MUC4 blockage, by the ErbB2 inhibitor AG825 (25 μM) or by the p38 inhibitor SB203580 (10 μM) in YY1 knockdown BXPC-3 cells transfected with the MMP10 promoter (*p* < 0.01) (Figure 5e). These results indicate that YY1 knockdown upregulates MMP10 by activating the MUC4/ErbB2/p38 MAPK signal transduction pathway.

### MUC4/ErbB2/p38 MAPK signal transduction-mediated MEF2C activation is involved in YY1 knockdown-induced MMP10 expression

To elucidate whether MEF2C, a direct target of p38 MAPK [28,29] and a direct activator of MMP10 transcription, was involved in the YY1 knockdown upregulated MMP10 gene transcription, luciferase activity assays were performed. The luciferase activity that was enhanced by YY1 knockdown was significantly inhibited by the MEF2C blockage (*p* < 0.001) (Figure 5f). Moreover, the luciferase activity of the MMP10 promoter in YY1 knockdown BXPC-3 cells was significantly decreased when the presumed MEF2C binding site (nucleotides −881 to −890) was mutated (*p* < 0.001) (Figure 5f). These results suggest that ErbB2/p38 MAPK signal transduction-mediated MEF2C activation maybe involved in YY1 knockdown-induced MMP10 expression.

## Discussion

In this study, we found that the expression of YY1 in PDACs was higher compared with their adjacent non-tumorous tissues and normal pancreas tissues. However, PDAC patients with high level overexpression of YY1 had better outcome than those with low level overexpression. YY1 overexpression suppressed, whereas YY1 knockdown enhanced, the proliferation, invasion and metastatic properties of BXPC-3 cells, both in vitro and in vivo. We also explored the potential mechanisms underlying the tumor suppression role of YY1 and found that YY1 suppresses invasion and metastasis of pancreatic cancer cells by downregulating MMP10 in a MUC4/ErbB2/p38/MEF2C-dependent mechanism. YY1 has an important role in many biological processes. As its name suggests, Yin-Yang 1 activates or inactivates gene expression depending on its interacting partners. Interestingly, its function in cancer remains controversial [3-5]. YY1 positively regulates the expression of several oncogenes, such as c-Myc and VEGF, and negatively regulates the expression of tumor suppressor genes, such as p53 and E-cadherin [30-33]. On the other hand, YY1 could suppress tumorigenesis by upregulating tumor suppressor genes such as HLJ1 and BRCA1, and inhibiting c-myc function by direct interaction [34-36]. Therefore, YY1 can act as both a tumor suppressor and an oncogene, depending on tissue context, interaction partners and downstream targets.

Several lines of evidence uncovered by the present study and our previous study indicate that YY1 may act as a tumor suppressor in PDAC. First, YY1 binds to the promoter of MUC4 and negatively regulates the expression of this well-known pancreatic cancer-related gene [23]. Second, we measured the expression levels of YY1 in 108 PDAC tissue samples with paired adjacent non-tumorous tissue and five normal pancreas tissue samples. We found that YY1 is highly expressed in PDAC tissues. However, interestingly, PDAC patients with high level overexpression of YY1 had better outcome than those with low level overexpression. We presumed that YY1 is not a causal factor of pancreatic cancer; however, during the development of pancreatic cancer, the human body provides feedback on increased expression of tumor suppressor gene YY1. Tumorigenesis is a complex biological process involving multi-factors, and is a competitive process of carcinogens and tumor suppressor factors. YY1, as a tumor suppressor, could not prevent cancer. However, YY1 could play a tumor suppressor role in inhibiting pancreatic cancer progression. Therefore, pancreatic cancer patients with high-level overexpression of YY1 show good prognosis. Thus, we could explain this seemingly contradictory phenomenon that the expression of YY1 in PDACs was higher compared with their adjacent non-tumorous tissues and normal pancreas tissues, but higher overexpression of YY1 was associated with and predicted a better outcome in PDAC patients. This hypothesis requires experimental validation. Finally, we found that overexpression of YY1 inhibits invasion and metastasis of BXPC-3 cells, both in vitro and in vivo, which would support the better clinical outcome of the PDAC patients with higher YY1. These findings indicate that YY1 negatively regulates MUC4 gene expression and might serves as a tumor suppressor in pancreatic cancer formation.

The membrane-bound mucin, MUC4, which is expressed as early as PanIN-1A but is not expressed in the healthy pancreas, and its membrane partner, the oncogenic receptor ErbB2, which is frequently overexpressed in pancreatic cancer as well as in PanINs, represent promising therapeutic targets [37,38]. Previous studies showed that MUC4 and ErbB2 interact physically and transduce signals intracellularly, thus promoting the migratory and metastatic potential of pancreatic cancer cells [24-26]. In this study, our DGE, qRT-PCR and western blotting experiments showed that YY1 overexpression downregulated, whereas YY1 knockdown upregulated, MUC4 expression in BXPC-3 cells. In addition, phosphorylation of ErbB2 decreased in YY1 overexpression BXPC-3 cells and increased in YY1 knockdown BXPC-3 cells. We then attempted to identify the cellular mechanisms and the intracellular signaling pathways under the control of MUC4 and ErbB2 partners. Our results showed that the p38 MAPK pathway was activated in YY1 knockdown BXPC-3 cells, but not the Erk1/2 or JNK MAPK pathways. Similarly, the Akt and FAK pathways were not significantly modified. These findings revealed that YY1 knockdown upregulates MUC4 expression, leading to activation of the ErbB2/p38 MAPK signal transduction pathway in BXPC-3 cells. In addition, we examined the correlation between YY1 and MUC4 expression in PDAC tissue samples by qRT-PCR. The results showed that YY1 expression was not statistically correlated with MUC4 expression. This result was not consistent with the in vitro result, which may reflect tissue heterogeneity and the complexity of the regulation of the MUC4 gene.

MMPs are proteinases that remodel the extracellular matrix and have previously been implicated in cancer metastasis and progression [39]. MMP-10 (also called stromelysin-2) belongs to the MMP family and its expression correlates closely with metastasis and poor prognosis in various human cancers [40]. In our study, MMP10 mRNA and protein expression were decreased in YY1 overexpression BXPC-3 cells and increased in YY1 knockdown BXPC-3 cells. In addition, YY1 knockdown significantly increased the luciferase activities of the MMP10 promoter and this effect could be suppressed by MUC4 blockage, or by inhibition of ErbB2 or p38 inhibitor, indicating that YY1 knockdown upregulated MMP10 by activating the MUC4/ErbB2/p38 MAPK signal transduction pathway. In the MMP10 blockage experiments, cell migration and invasion were significantly reduced when the MMP10 activity was blocked by siRNA in YY1 knockdown BXPC-3 cells, indicating that YY1 knockdown BXPC-3 cells with an activated p38 MAPK pathway results in increased MMP10 expression, thus increasing the migratory and invasive abilities of the cells. Moreover, in PDAC tissue samples, YY1 mRNA expression levels were statistically negatively correlated with MMP10 expression levels, which was consistent with the in vitro result.

MEF2C is a MADS domain transcription factor that regulates the development and differentiation of many tissue types [41]. It is phosphorylated by p38 MAPK in myocytes and macrophages [28,29]. In addition, previous studies indicated that MEF2C promotes the transcription of MMP10 by binding to the upstream promoter [27]. In our study, the results showed that YY1 overexpression downregulated, whereas YY1 knockdown upregulated, MEF2C expression in BXPC-3 cells. Luciferase activity assays showed that the MMP10 promoter activity that was enhanced by YY1 knockdown was significantly decreased by MEF2C blockage. Moreover, mutating the MEF2C-binding site in the MMP10 luciferase reporter completely abolished the effect of YY1 knockdown, indicating that MUC4/ErbB2/p38 MAPK signal transduction-mediated MEF2C activation is involved in YY1 knockdown-induced MMP10 expression.

## Conclusions

**Figure 6:**
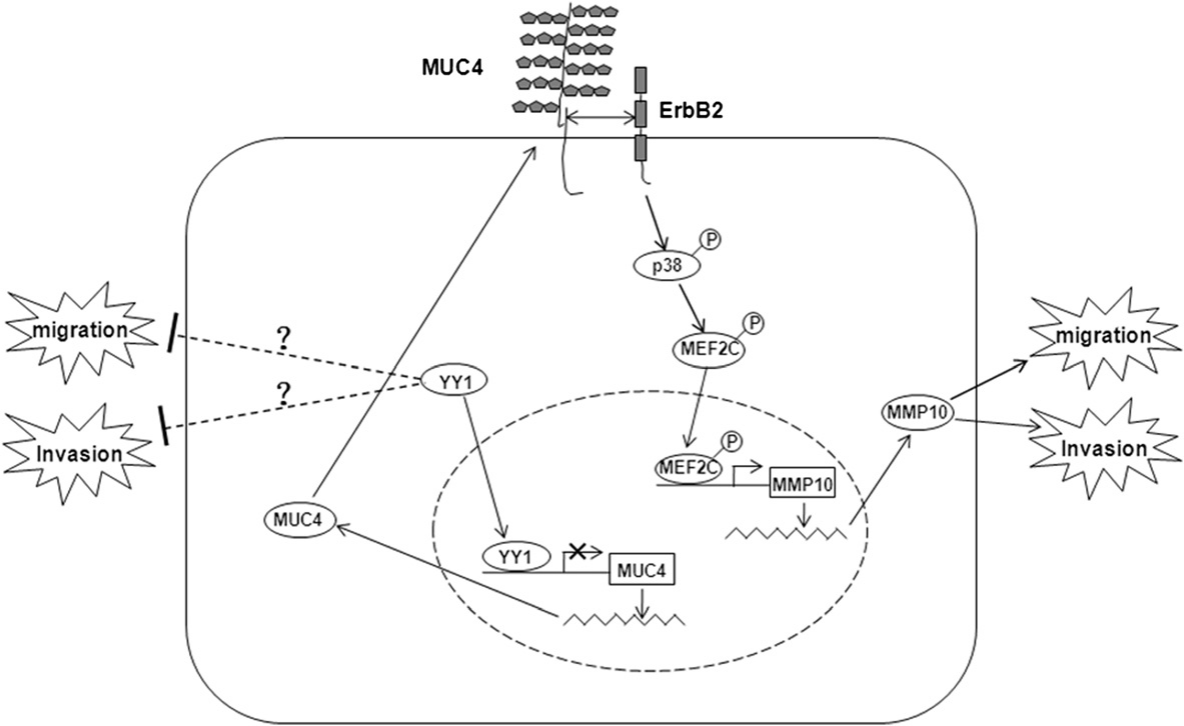
**Schematic representation of the roles of YY1 on the migrating and invasive properties of BXPC-3 cells.** YY1 suppresses invasion and metastasis of BXPC-3 cells by downregulating MMP10 in a MUC4/ErbB2/p38/MEF2C-dependent mechanism. “?” represents other undiscovered mechanisms that might be involved.

In summary, YY1 is highly expressed in all PDAC. Higher overexpression of YY1, however, was associated with, and predicted, a better outcome in PDAC patients. The YY1 high-level overexpression suppresses invasion and metastasis of pancreatic cancer cells by downregulating MMP10 via a MUC4/ErbB2/p38/MEF2C-dependent mechanism (Figure 6). Although other undiscovered mechanisms maybe involved in the YY1-mediated tumor suppression role, the present study suggested that YY1 high-level overexpression (≥1.159) plays a negative role; i.e., acts as a tumor suppressor in PDAC. YY1 expression levels may represent a valuable diagnostic and prognostic marker of PDAC.

## Methods

### Patients and pancreatic tissues

Pancreatic tissue samples were obtained from 108 patients (average age 61.0 years; range 25–82 years) who underwent pancreaticoduodenectomy for PDAC at the first Affiliated Hospital of Nanjing Medical University, China, between 2006 and 2012. No chemotherapy or radiation therapy was administered before tumor excision. Written informed consents were obtained from all patients undergoing surgery and the Ethics Committees of the first Affiliated Hospital of Nanjing Medical University approved the study. One hundred and eight pairs of tumorous and non-tumorous tissue samples from these patients were collected during surgery. In addition, samples of normal pancreas tissue were obtained from five patients who underwent partial pancreatectomy for benign pancreatic tumors. Immediately (within 5 min) upon surgical removal, each tissue sample was cut in two, one was snap-frozen in liquid nitrogen until use (for RNA or protein extraction) and the other was fixed in 5% formalin and embedded in paraffin after 24 hr (for immunohistochemistry). A pathologist examined all tissue samples histologically to confirm the diagnosis.

The 108 PDAC patients were followed up regularly until 31 May 2013. Patients’ overall survival was defined as the time between surgery and death or the last follow-up date. None of the 108 selected patients died within one month after surgery. The clinicopathological and serological characteristics of the 108 patients are summarized in Table 1. Tumor differentiation was assigned according to the World Health Organization criteria [42]. TNM staging was based on the sixth edition of the American Joint Committee on Cancer (AJCC) guidelines [43].

### Quantitative RT-PCR (qRT-PCR)

Total RNAs were prepared using the TRIzol reagent (Life Technologies, Carlsbad, CA, USA), according to the manufacturer’s protocol. After spectrophotometric quantification, 1 μg of total RNA was used for reverse transcription (RT) in a final volume of 20 μl with an iScript cDNA Synthesis Kit (Bio-Rad, Hercules, CA, USA) according to the manufacturer’s instructions. Quantitative PCR was performed using TaqMan Gene Expression Assays (Life Technologies) in a StepOne Plus Real-time PCR System (Life Technologies). The reactions were performed in a volume of 10 μl containing 1 μl diluted cDNA, 20 × TaqMan Gene Expression Assay Mix and 2 × TaqMan Universal PCR Master Mix. The thermal cycling conditions comprised an initial denaturation step at 95°C for 10 min, 40 cycles at 95°C for 15 s and 60°C for 1 min. The TaqMan Gene Expression Assay Mixes used for YY1, MMP10, TIMP2, MUC4 and MEF2C had the product numbers Hs00231533_m1, Hs00233987_m1, Hs00234278_m1, Hs00366414_m1 and Hs00231149_m1, respectively. Human 18S rRNA (product number Hs099999901_s1) or GAPDH (product number Hs02758991_g1) were used to calibrate the original concentration of mRNA. The relative gene expression was calculated by the subtraction of the Ct value of YY1 (target) and 18S rRNA or GAPDH (control) genes by the 2^-ΔCT^ method [44]. Each quantitative PCR was performed in triplicate and independently repeated three times.

### Tissue microarrays and immunohistochemistry

To verify the YY1 expression in PDAC tissues detected by qRT-PCR, tissue microarrays (TMA) containing five normal pancreas tissue samples and 108 pairs of PDAC samples and their corresponding non-tumorous tissues (duplicate 2 mm tissue cores for each PDAC) were constructed by Shanghai Outdo Biotech co., Ltd (Shanghai, China).

Immunohistochemistry was performed as follows. 4-μm-thick TMA sections were cut and mounted on poly-L-lysine-coated glass slides. Slides were deparaffinized in xylene, rehydrated in graded alcohol and washed in tap water. Endogenous peroxidase was blocked by incubating the sections in 3% H_2_O_2_ for 5 min. Antigen retrieval was then performed for 20 min in 10 mmol/L sodium citrate buffer (pH 6.0) heated at 95°C in a microwave oven, followed by a blocking step with 5% normal goat serum for 10 min at room temperature. After blocking, the sections were incubated at 4°C overnight with anti-YY1 polyclonal antibody (Santa Cruz Biotechnology, Santa Cruz, CA, USA, 1:150 dilution), followed by incubation with a secondary antibody (goat anti-rabbit/horseradish peroxidase, Santa Cruz Biotechnology, 1:200 dilution) at room temperature for 30 min. Finally, the sections were developed with diaminobenzidine substrate for about 5 min, and counterstained with hematoxylin. Two authors (Yi Zhu and Jing-Jing Zhang) evaluated the immunohistochemical data for YY1. Expression levels were evaluated according to the staining intensity (0 for absent, 1 for weak, 2 for moderate and 3 for strong staining) and proportion of positive cells (0 for <10%, 1 for 10 to <50%, and 2 for ≥50% of cells). The sum of intensity and proportion scores were then used to determine the immunoreactivity.

### Cell lines and cell culture

Six human PDAC cell lines (BXPC-3, PANC-1, HPAC, CFPAC-1, SW1990 and COLO-357) were purchased from the Shanghai Cell Bank (Shanghai, China). The cells were grown in Dulbecco’s modified Eagle’s medium (DMEM) (Life Technologies) supplemented with 10% fetal calf serum (FBS) (Wisent Inc., Montreal, Qc, Canada), 10 mM HEPES (Sigma, St Louis, MO, USA), 2 mM L-glutamine (Sigma), 1 mM pyruvate sodium (Sigma), 100 units/ml penicillin (Life Technologies), and 100 μg/ml streptomycin (Life Technologies) at 37°C in a humidified atmosphere containing 95% air and 5% CO_2_.

### Preparation of YY1 overexpression BXPC-3 cells

Sunbio Medical Biotechnology Co., Ltd (Shanghai, China) constructed the YY1-overexpression lentiviruses. Briefly, the full-length coding region of human YY1 was subcloned into pCDH-CMV-MCS-EF1-Puro vector (System Biosciences, Mountain View, CA, USA) and verified by sequencing. The verified recombinant vector and the pPACKH1 packaging plasmid mix (System Biosciences) were co-transfected into 293T cells using the Lipofectamine 2000 reagent (Life Technologies). The supernatant of the cultured 293T cells was collected to infect BXPC-3 cells. The pCDH-CMV-MCS-EF1-Puro vector was used to package the virus and infect BXPC-3 cells as a control. Stable cell lines (BXPC-Vector and BXPC-YY1) were selected by culturing in media containing 5 μg/ml puromycin (Sigma). YY1 expression was confirmed by qRT-PCR and Western blot.

### Preparation of YY1 knockdown BXPC-3 cells

Sunbio Medical Biotechnology Co., Ltd (Shanghai, China) constructed the YY1-knockdown lentiviruses. The target DNA sequences corresponding to YY1-shRNA were detailed in a previous report [45]. The selected sequences were as follows: Forward oligonucleotide, 5′ccggGGGAGCAGAAGCAGGTGCAGATctcgagATCTGCACCTGCTTCTGCTCCCtttttg-3′; Reverse oligonucleotide: 5′ aattcaaaaaGGGAGCAGAAGCAGGTGCAGATctcgagATCTGCACCTGCTTCTGCTCCC-3′, with AgeI and EcoRI restriction enzyme sites (5′ and 3′ ends, respectively). The target DNA was subcloned into pLKO.1-TRC Cloning Vector (Addgene, Cambridge, MA, USA). The verified recombinant vector plasmid (pLKO.1/YY1-shRNA), packaging plasmid psPAX2 (Addgene) and envelope plasmid pMD2.G (Addgene) were co-transfected into 293T cells using the Lipofectamine 2000 reagent. The supernatant of the cultured 293T cells was collected to infect BXPC-3 cells. The pLKO.1-scramble shRNA (Addgene, Negative control vector containing scramble shRNA insert) was used to package the virus and infect BXPC-3 cells as a control. Stable cell lines (BXPC-Scramble shRNA and BXPC-YY1 shRNA) were selected by culturing in media containing 5 μg/ml puromycin (Sigma). qRT-PCR and western blotting confirmed the YY1 expression.

### Western blotting

Pancreatic cancer cells or pancreas tissue were lysed in ice-cold lysis buffer containing the following reagents: 50 mM Tris–HCl pH 7.4; 1% NP-40; 150 mM NaCl; 1 mM EDTA; 1 mM PMSF and complete proteinase inhibitor mixture (1 tablet per 10 ml, Roche Diagnostics GmbH, Mannheim, Germany). The DC protein assay kit (Bio-Rad) was used to quantify the protein concentration in the cell lysate. Protein aliquots were electrophoresed using 12% SDS-PAGE and transferred to a PVDF membrane (Bio-Rad). Nonspecific protein interactions were blocked by incubation in 5% nonfat dry milk in TBST buffer [20 mM Tris–HCl, 150 mM NaCl, 0.1% Tween 20 (pH 7.6)] at room temperature for 1 h and then washed with TBST. Membranes were then incubated at 4°C overnight with primary antibodies in fresh blocking buffer. The antibodies to YY1 or GAPDH were from Santa Cruz. The antibodies to MUC4 and MEF2C were from Abcam (Cambridge, MA, USA). Primary antibodies to phospho-HER2/ErbB2 (Tyr1248), HER2/ErbB2, phospho-Erk1/2 (Thr202/Tyr204), Erk1/2, phospho-SAPK/JNK (Thr183/Tyr185), SAPK/JNK, phospho-p38 MAPK (Thr180/Tyr182), p38 MAPK, phospho-Akt (Thr308), phospho-Akt (Ser473), Akt, phospho-FAK (Tyr397) and FAK were from Cell Signaling Technology (Danvers, MA, USA). The antibody to MMP10 was from Epitomics-an Abcam Company (Burlingame, CA, USA). The blots were then washed and incubated with HRP-conjugated secondary antibodies (Cell Signaling) for 1 h at room temperature. The bands were visualized with Immobilon Western Chemilum HRP substrate (Merck Millipore, Darmstadt, Germany) using the Fluorchem E System (ProteinSimple, Santa Clara, CA, USA). Prestained markers (Thermo Scientific, Rockford, IL, USA) were used as internal molecular weight standards. Each blot was independently repeated three times.

### Cell Count Kit-8 (CCK-8) assay

To detect cell proliferation, we used the CCK-8 assay (DOJINDO, Japan), according to the manufacturer’s protocol. Cells were seeded into 96-well plates at 2 × 10^3^/well. At the same time of each day, 10 μl CCK-8 was added to each well, which contained 100 μl medium. After incubation for 3 hours, the absorbance at 450 nm of each well was measured using a microplate reader. Each sample had four duplicate wells and was independently repeated three times.

### Cell migration and invasion assays

Cell migration and invasion were assessed using 24 well Millicell Hanging Cell Culture Inserts (PET membranes with 8 μm pores, Merck Millipore). For invasion assays, the upper surface of each insert was coated with Matrigel (BD Bioscience Pharmingen), following manufacturer’s protocol. Briefly, 10% (v/v) fetal bovine serum was used as the chemoattractant in the lower chamber. 5 × 10^4^ cells were seeded in the upper chamber and incubated at 37°C, with migration assessed at 24 hours and invasion at 48 hours. Non-migrating or non-invading cells were removed from the upper surface of the membrane by wiping with cotton-tipped swabs. Cells on the lower surface of the membrane were stained with 0.1% crystal violet for 10 minutes and photographed. The stained cells were then soaked in 33% acetic acid and oscillated for 10 min. The absorbance of 33% acetic acid containing crystal violet was then assessed using a microplate reader (Tecan, Shanghai, China) at a 570 nm wavelength. The OD (optical density) value indirectly reflected the number of penetrated cells. OD values for triplicate membranes were reported as the mean ± SD and the experiments were repeated three times.

In the MMP10 blockage experiments, YY1 knockdown BXPC-3 cells were transfected with MMP10 siRNA or negative control siRNA (Life Technologies, final concentration of 30 nM) using Lipofectamine 2000, according to the manufacturer’s protocol. 12 h after transfection, cells were seeded in the upper chamber and incubated. Cell migration and invasion assays were performed as described above.

### In vivo tumor growth and metastasis study

Four-week-old female nude mice (BALB/cA-nu) were purchased from the Shanghai Experimental Animal Center (Chinese Academy of Sciences, Shanghai, China). Thirty-two mice were randomly divided into four groups. For the xenograft subcutaneous implant tumor model, BXPC-Scramble shRNA and BXPC-YY1 shRNA cells were bilaterally injected subcutaneously into the flank region of the mice (1.5 × 10^6^ cells/100 μl per flank), respectively. Bidimensional tumor measurements were taken with calipers once weekly. The tumor volume was calculated using the formula (width^2^ × length)/2. For the tail vein metastasis model, BXPC-Vector and BXPC-YY1 cells (1.5 × 10^6^ cells/100 μl) were separately injected into the tail vein of each mouse. Four weeks later, the mice were euthanized and subcutaneous tumors, lungs and livers were removed and fixed in 4% paraformaldehyde. Deparaffinized sections were stained using HE stain by staff of the Pathology Department of the first Affiliated Hospital of Nanjing Medical University. The histomorphology of the tumor samples and extent of metastasis in the lung and liver were evaluated.

### Digital gene expression (DGE) sequencing

Six micrograms of total RNA were extracted from BXPC-Scramble shRNA and BXPC-YY1 shRNA cells as described in section 2.2. Quality and quantity analysis of total RNA, DGE library preparation, and sequencing were carried out at Huada Genomics Co., Ltd. (Shenzhen, China). mRNA was purified using Oligo (dT) magnetic beads adsorption, and then Oligo(dT) was used as primer to synthesize the first and second-strand cDNA. The 5′ ends of tags could be generated by two types of endonuclease: NlaIII or DpnII. The bead-bound cDNA was subsequently digested with restriction enzyme NlaIII, which recognizes and cuts off the CATG sites. The fragments, except from the 3′ cDNA fragments connected to Oligo(dT) beads were washed away and the Illumina adaptor1 was ligated to the sticky 5′ end of the digested bead-bound cDNA fragments. The junction of Illumina adaptor 1 and CATG site is the recognition site of MmeI, an endonuclease with separate recognition sites and digestion sites. It cuts 17 bp downstream of the CATG site, producing tags with adaptor 1. After removing 3′ fragments with magnetic beads precipitation, Illumina adaptor 2 was ligated to the 3′ ends of the tags, producing tags with different adaptors of either end to form a tag library. After linear PCR amplification, fragments were purified by PAGE Gel electrophoresis. Finally, two DGE sequence libraries were sequenced using Illumina HiSeq 2000. The raw sequences were filtered to leave clean tags and then mapped to the transcriptome, to be used as reference sequences containing all the possible clean tags containing CATG and 17 base-length sequences of the reference gene sequences. The number of unambiguous clean tags for each gene was calculated and then normalized to the number of transcripts per million clean tags (TPM) [46,47]. A rigorous algorithm was developed to identify differentially expressed genes between the two groups of cells, using method described previously [48]. The *p*-value corresponds to differential gene expression test. The false discovery rate (FDR) was used to determine the threshold of the *p* value in multiple tests and analysis. We use FDR ≤ 0.001 and the absolute value of log2 ratio ≥ 1 as the threshold to judge the significance of gene expression differences [49]. We perpormed two biological replicates of the DGE sequencing and took the average of the two before differential gene expression analysis. For gene expression profiling analysis, we performd gene ontology (GO) enrichment analysis for functional significance using a hypergeometric test to map all DEGs to terms in GO database, looking for significantly enriched GO terms in DEGs compared with the genome background.

### Construction of reporter gene plasmids

A luciferase reporter construct containing the human MMP10 promoter (−1000/-1, upstream of translation initiation sites, TIS) was prepared using the pGL3-basic vector (Promega, Madison, WI, USA). The GenScript Biotechnology Co., Ltd (Nanjing, China) synthesized a DNA fragment of MMP10 promoter region (including the sites of restriction enzymes). The DNA fragment was subcloned into the KpnI and XhoI sites of the pGL3-basic vector to construct pGL3-MMP10-promoter (pMMP10) recombinant plasmid and then confirmed by sequencing.

The mutant construct containing the MMP10 promoter in which the presumed MEF2C binding site (nucleotides −881 to −890) was mutated from TTAAAAAACA to TTAGGGGACA was also constructed. The construct was named pMMP10-MEF2C-M.

### Cell transient transfection and luciferase assay

Transfections were performed using Lipofectamine 2000, according to the manufacturer’s protocol. Cells were plated into 12-well cell culture plates (2 × 10^5^/well) 1 day before transfection. Each transfection was performed using 1 μg luciferase reporter construct (pMMP10 or pMMP10-MEF2C-M) plus 2.5 ng Renilla luciferase reporter vector, pRL-SV40 as an internal control (Promega). 48 h after transfection, cells were washed with PBS and lysed using 1× passive lysis buffer. Firefly and Renilla luciferase activities were measured with a GloMax-20/20 luminometer (Promega) using the Dual-Luciferase Reporter Assay System (Promega). Firefly luciferase activity was normalized to the Renilla luciferase activity. Each experiment was performed in triplicate and independently repeated three times.

For the MUC4 or MEF2C blockage experiments, cells were transfected with 1 μg pMMP10, 2.5 ng pRL-SV40 plus MUC4 siRNA, MEF2C siRNA or negative control siRNA (Life Technologies, final concentration of 30 nM). 48 h after transfection, the luciferase activity was analyzed as described above.

For the signal transduction pathways blockage experiments, cells were transfected with 1 μg pMMP10 plus 2.5 ng pRL-SV40. At 12 h after transfection, the medium was replaced and cells were treated for 24 h with 25 μM AG825 (Merck Millipore), 10 μM SB203580 (Cell Signaling Technology) or equivalent amounts of DMSO vehicle (Sigma) as a control. The luciferase activity was then analyzed as described above.

### Statistical analysis

Statistical analysis was performed using the SPSS software (Version 15.0). Quantitative data were presented as mean ± SD. Differences in the mean of two samples were analyzed by Student’s *t* test. Differences in YY1 mRNA or protein levels between PDACs and their adjacent non-tumorous tissues were analyzed using the Mann–Whitney U test. Correlations between YY1 mRNA levels and protein levels were analyzed by the Spearman rank correlation test. Correlations between YY1 mRNA levels and MUC4 or MMP10 mRNA levels were also analyzed by the Spearman rank correlation test. Correlations between YY1 mRNA expression and each clinicopathological or serological variable were analyzed by the Mann–Whitney U or Kruskal-Wallis test. Receiver operating characteristic (ROC) curve analysis determined the YY1 expression level cut-off value for survival analysis [50-52]. Survival distributions and overall survival rates were determined using the Kaplan-Meier method, and the significance of differences between survival rates was calculated by the Log-rank test. The variables that showed a statistically significant prognostic value in univariate analyses were entered into a multivariate model and excluded for *p* > 0.10. A stepwise selection of factors was applied to the multivariate Cox regression model to identify independent prognostic factors for overall survival. In addition, owing to the small number of mice used, the data obtained using the tumor models were analyzed by Fisher’s exact test. All statistical tests were two-tailed exact tests with a *p* < 0.05 considered significant.

## Abbreviations

CCK-8: Cell count kit-8; CI: Confidence interval; DEGs: Differentially expressed genes; DGE: Digital gene expression; DMEM: Dulbecco’s modified Eagle’s medium; FBS: Fetal calf serum; FDR: False discovery rate; GO: Gene ontology; HR: Hazard ratio; IHC: Immunohistochemistry; OD: Optical density; PDAC: Pancreatic ductal adenocarcinoma; qRT-PCR: Quantitative reverse transcription polymerase chain reaction; ROC: Receiver operating characteristic; RT: Reverse transcription; TMA: Tissue microarrays; TPM: Transcripts per million clean tags; YY1: Yin Yang-1.

## Competing interests

The authors declare that they have no competing interests.

## Authors’ contributions

JJZ and YZ carried out the studies, participated in the experimental design, statistical analysis and drafted the manuscript. KLX carried out the in vivo studies and participated in the statistical analysis. YPP and JQT participated in the plasmid construction and luciferase reporter assays. JT and ZL participated in the qRT-PCR and western blotting. ZKX, CCD, ZYQ, KRJ, JLW, WTG and QD participated in the sample collection, patients’ follow-up and statistical analysis. YM conceived of the study, and participated in its design and coordination and helped to draft the manuscript. All authors read and approved the final manuscript.

## Supplementary Material

Additional file 1: Table S1 Correlations between YY1 mRNA expression and clinicopathological and serological variables (n = 108). Click here for file

Additional file 2: Table S2 Univariate analysis of prognostic factors in PDAC patients (n = 108). Click here for file

Additional file 3: Table S3 Differentially expressed genes between YY1 knockdown BXPC-3 cells and control cells. Click here for file

Additional file 4: Figure S1 Validation of effects of YY1 on gene expression. Expression levels of selected genes (MMP10, TIMP2, MUC4 and MEF2C) from DGE sequencing were studied by qRT-PCR in BXPC-Scramble shRNA and BXPC-YY1 shRNA cells. Click here for file

Additional file 5: Figure S2 GO functional enrichment analysis for differentially expressed genes (DEGs). All DEGs were assigned functionally into three groups: (1) biological process; (2) cellular component; (3) molecular function. Within biological process, cellular process and metabolic process represented the most abundant GO terms. Most DEGs that corresponded to cellular component were involved in cell and cell part. Binding and catalytic activity were the most prevalent in molecular function. Click here for file

Additional file 6: Figure S3 Effects of YY1 on FAK and Akt signaling pathways. Western blotting was carried out for phospho-FAK, FAK, phospho-Akt and Akt in BXPC-YY1, BXPC-YY1 shRNA and their respective controls (BXPC-Vector and BXPC-Scramble shRNA). GAPDH was used as the internal control. Click here for file
