# Supplementary material for: Yin Yang-1 suppresses invasion and metastasis of pancreatic ductal adenocarcinoma by downregulating MMP10 in a MUC4/ErbB2/p38/MEF2C-dependent mechanism
Source: Mol Cancer. 2014 May 29;13:130. doi: 10.1186/1476-4598-13-130 (PMC4047260; doi:10.1186/1476-4598-13-130)
Supplement: Additional file 1: Table S1 — Correlations between YY1 mRNA expression and clinicopathological and serological variables (n = 108). [file 1476-4598-13-130-S1.doc]

**Table S1.** Correlations between YY1 mRNA expression and clinicopathological and serological variables (n=108).

| **Variable** | **Case** | **YY1** | | |
| --- | --- | --- | --- | --- |
|  |  | **Mean** | **SD** | ***p*** |
| Gender |  |  |  | 0.068 |
| Male | 61 | 0.880 | 0.925 |  |
| Female | 47 | 1.189 | 1.104 |  |
| Age (y) |  |  |  | **0.022^*^** |
| < 60 | 47 | 1.188 | 0.994 |  |
| ≥ 60 | 61 | 0.881 | 1.016 |  |
| Location of tumor |  |  |  | 0.103 |
| Head | 72 | 1.122 | 1.060 |  |
| Body and tail | 36 | 0.800 | 0.889 |  |
| Size of tumor (cm) |  |  |  | 0.325 |
| ≤ 2 | 22 | 1.232 | 1.138 |  |
| >2 | 86 | 0.959 | 0.979 |  |
| Differentiation |  |  |  | **<0.001^*^** |
| Well | 15 | 2.115 | 1.325 |  |
| Moderate | 83 | 0.890 | 0.859 |  |
| Poor | 10 | 0.393 | 0.418 |  |
| Nerve infiltration |  |  |  | 0.847 |
| No | 38 | 0.949 | 0.894 |  |
| Yes | 70 | 1.055 | 1.085 |  |
| TNM staging |  |  |  | **0.008^*^** |
| IA+IB | 15 | 1.141 | 1.065 |  |
| IIA | 33 | 1.187 | 0.968 |  |
| IIB | 46 | 1.066 | 1.095 |  |
| III+IV | 14 | 0.301 | 0.361 |  |
| Serum CA19-9 (kU/L) |  |  |  | 0.155 |
| ≤ 39 | 31 | 1.182 | 1.027 |  |
| > 39 | 77 | 0.947 | 1.007 |  |
| Serum CA50 (kU/L) |  |  |  | 0.673 |
| ≤ 25 | 49 | 1.064 | 1.059 |  |
| > 25 | 59 | 0.974 | 0.982 |  |
| Serum CEA (μg/L) |  |  |  | 0.342 |
| ≤ 4.3 | 64 | 1.075 | 1.031 |  |
| > 4.3 | 44 | 0.926 | 0.993 |  |

^*^ *p* < 0.05.
