# Supplementary material for: Yin Yang-1 suppresses invasion and metastasis of pancreatic ductal adenocarcinoma by downregulating MMP10 in a MUC4/ErbB2/p38/MEF2C-dependent mechanism
Source: Mol Cancer. 2014 May 29;13:130. doi: 10.1186/1476-4598-13-130 (PMC4047260; doi:10.1186/1476-4598-13-130)
Supplement: Additional file 2: Table S2 — Univariate analysis of prognostic factors in PDAC patients (n = 108). [file 1476-4598-13-130-S2.doc]

**Table S2. Univariate analysis of prognostic factors in PDAC patients (n=108).**

| **Variable** | **Cases** | **Events** | **Mean survival (months)** | **HR** ^a^ (**95% CI** ^b^) | ***p*** |
| --- | --- | --- | --- | --- | --- |
| Gender |  |  |  |  | 0.507 |
| Male | 61 | 47 | 16.209 | 1 |  |
| Female | 47 | 37 | 19.699 | 0.863 (0.559-1.333) |  |
| Age (y) |  |  |  |  | 0.177 |
| < 60 | 47 | 35 | 21.381 | 1 |  |
| ≥ 60 | 61 | 49 | 15.254 | 1.350 (0.873-2.089) |  |
| Location of tumor |  |  |  |  | **0.026^*^** |
| Head | 72 | 52 | 20.784 | 1 |  |
| Body and tail | 36 | 32 | 12.263 | 1.654 (1.061-2.578) |  |
| Size of tumor (cm) |  |  |  |  | 0.356 |
| ≤ 2 | 22 | 67 | 20.162 | 1 |  |
| >2 | 86 | 17 | 17.301 | 1.286 (0.754-2.194) |  |
| Differentiation |  |  |  |  | **<0.001^*^** |
| Well | 15 | 8 | 35.020 | 1 |  |
| Moderate | 83 | 66 | 15.803 | 2.535 (1.204-5.340) | 0.014^*^ |
| Poor | 10 | 10 | 5.800 | 7.584 (2.903-19.816) | <0.001^*^ |
| Nerve infiltration |  |  |  |  | 0.697 |
| No | 38 | 29 | 21.163 | 1 |  |
| Yes | 70 | 55 | 16.420 | 1.095 (0.694-1.726) |  |
| TNM staging |  |  |  |  | **0.008^*^** |
| IA+IB | 15 | 12 | 24.448 | 1 |  |
| IIA | 33 | 25 | 17.483 | 1.372 (0.684-2.752) | 0.373 |
| IIB | 46 | 34 | 18.785 | 1.507 (0.776-2.924) | 0.226 |
| III+IV | 14 | 13 | 6.982 | 3.715 (1.657-8.329)) | 0.001^*^ |
| Serum CA19-9 (kU/L) |  |  |  |  | **0.014^*^** |
| ≤ 39 | 31 | 16 | 29.025 | 1 |  |
| > 39 | 77 | 68 | 14.569 | 1.989 (1.150-3.438) |  |
| Serum CA50 (kU/L) |  |  |  |  | 0.064 |
| ≤ 25 | 49 | 30 | 24.091 | 1 |  |
| > 25 | 59 | 54 | 14.459 | 1.529 (0.975-2.398) |  |
| Serum CEA (μg/L) |  |  |  |  | **0.033^*^** |
| ≤ 4.3 | 64 | 44 | 22.116 | 1 |  |
| > 4.3 | 44 | 40 | 12.117 | 1.607 (1.039-2.484) |  |
| YY1 |  |  |  |  | **<0.001^*^** |
| Low level overexpression | 72 | 63 | 10.768 | 1 |  |
| High level overexpression | 36 | 21 | 31.245 | 0.310 (0.182-0.527) |  |

^a^ HR, hazard ratio; ^b^ 95% CI, 95% confidence interval; ^*^ *p* < 0.05.
