# Supplementary figures and images for: Yin Yang-1 suppresses invasion and metastasis of pancreatic ductal adenocarcinoma by downregulating MMP10 in a MUC4/ErbB2/p38/MEF2C-dependent mechanism
Source: Mol Cancer. 2014 May 29;13:130. doi: 10.1186/1476-4598-13-130 (PMC4047260; doi:10.1186/1476-4598-13-130)

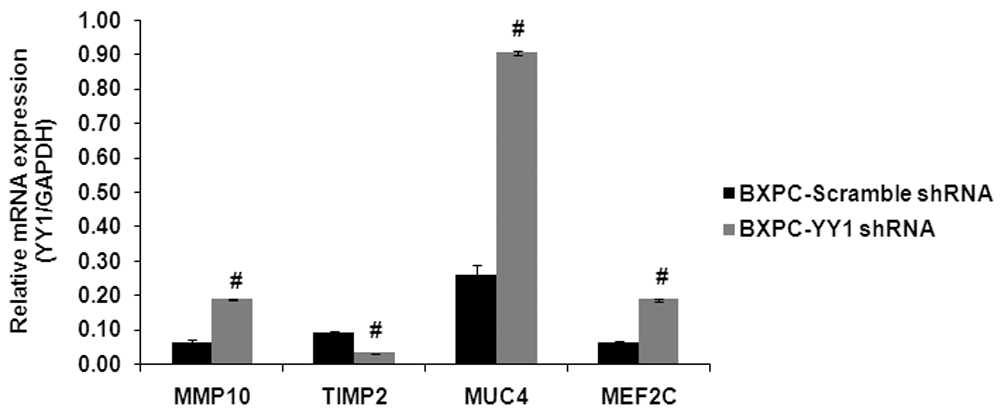

Supplement: Additional file 4: Figure S1 — Validation of effects of YY1 on gene expression. Expression levels of selected genes (MMP10, TIMP2, MUC4 and MEF2C) from DGE sequencing were studied by qRT-PCR in BXPC-Scramble shRNA and BXPC-YY1 shRNA cells. [file 1476-4598-13-130-S4.tiff]

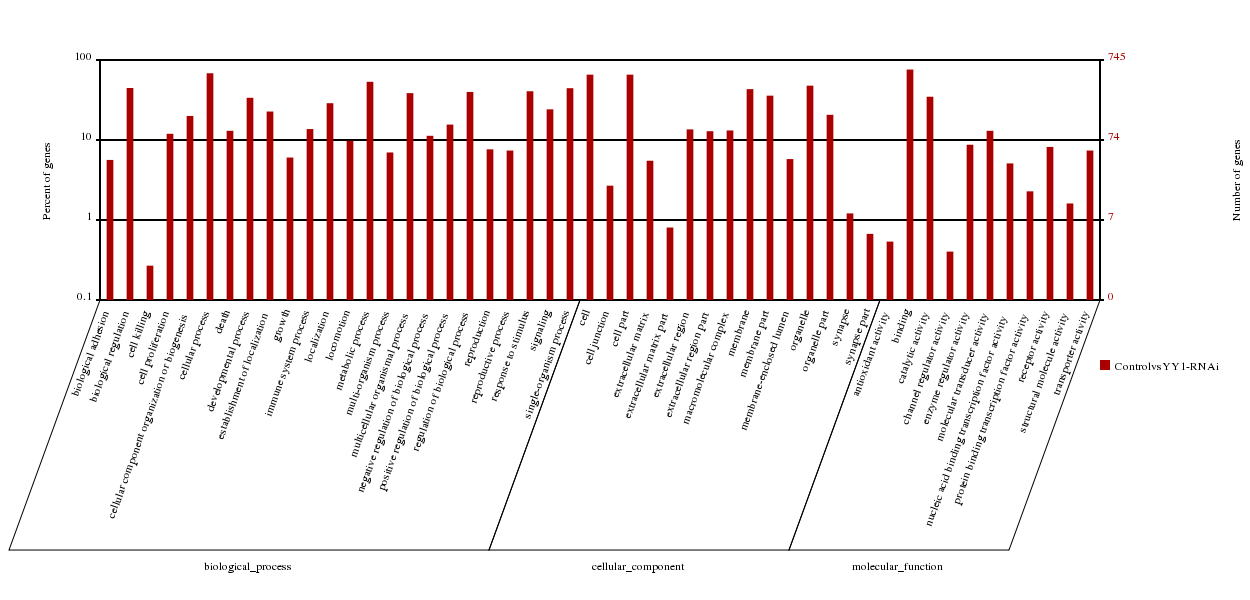

Supplement: Additional file 5: Figure S2 — GO functional enrichment analysis for differentially expressed genes (DEGs). All DEGs were assigned functionally into three groups: (1) biological process; (2) cellular component; (3) molecular function. Within biological process, cellular process and metabolic process represented the most abundant GO terms. Most DEGs that corresponded to cellular component were involved in cell and cell part. Binding and catalytic activity were the most prevalent in molecular function. [file 1476-4598-13-130-S5.png]

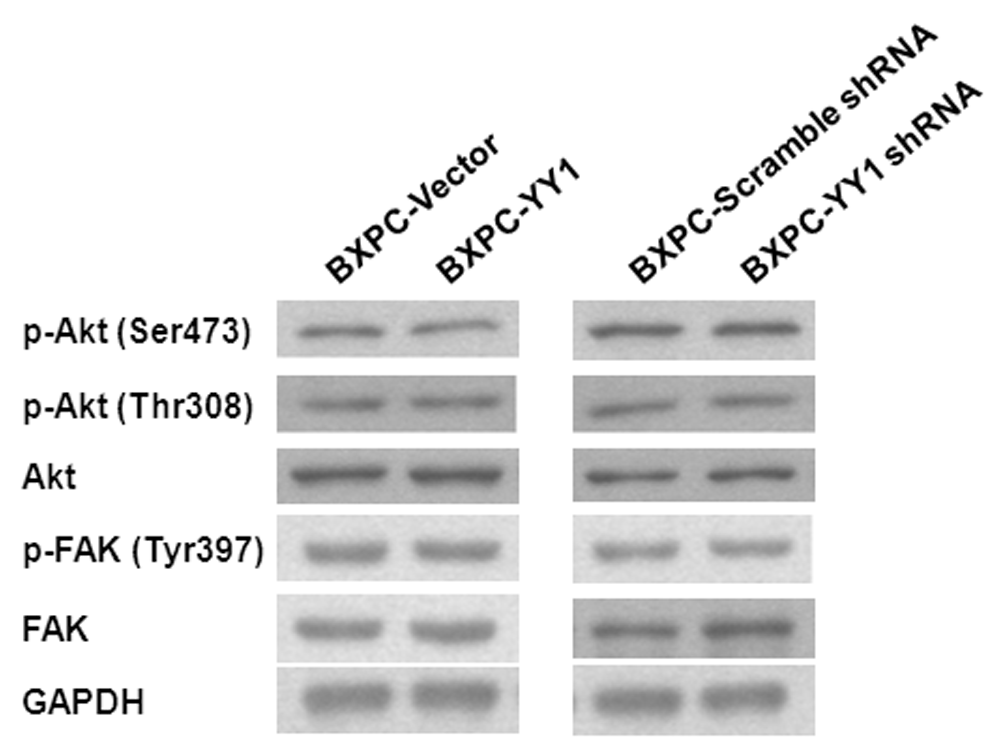

Supplement: Additional file 6: Figure S3 — Effects of YY1 on FAK and Akt signaling pathways. Western blotting was carried out for phospho-FAK, FAK, phospho-Akt and Akt in BXPC-YY1, BXPC-YY1 shRNA and their respective controls (BXPC-Vector and BXPC-Scramble shRNA). GAPDH was used as the internal control. [file 1476-4598-13-130-S6.tiff]
